# Patient-Centred Care for Multimorbid Patients: A Scoping Review

**DOI:** 10.3390/jcm15103774

**Published:** 2026-05-14

**Authors:** Ine Van den Wyngaert, Bert Vaes, Nicolas Delvaux, Gijs Van Pottelbergh

**Affiliations:** Academic Centre for General Practice, Department of Public Health and Primary Care, KU Leuven, Kapucijnenvoer 7 Blok g—Bus 7001, 3000 Leuven, Belgium; bert.vaes@kuleuven.be (B.V.); nicolas.delvaux@kuleuven.be (N.D.); gijs.vanpottelbergh@kuleuven.be (G.V.P.)

**Keywords:** patient-centred, multiple chronic conditions, multimorbidity, primary care

## Abstract

**Background/Objectives:** The Belgian healthcare system is currently organised from a single-disease point of view, which poses a unique challenge for patients suffering from two or more long-term physical and/or mental conditions (multimorbidity) and their healthcare providers. To search for strategies that respond to the constantly changing medical landscape and the complexity of multimorbid care. Patient-centred care programmes for multimorbid patients in primary care were investigated and their intervention elements were assessed as to whether or not they have a positive effect on the Triple Aim. **Methods:** A scoping review was performed and reported following the PRISMA-ScR guidelines. Online databases (PubMed, Cochrane, Embase) were used to identify papers published between January 2000 and August 2023, supplemented by reference tracking and a manual search in the grey literature. Studies were included if they assessed the efficacy of patient-centred intervention in primary care for multimorbid adult patients. **Results:** From the 7020 papers identified, 39 were selected and included in the review. Interventions took place at three levels (patient, professional and organisational). The efficacy of the studies included was heterogeneous. The different intervention elements had a more frequent positive effect on healthcare experience than on health status and behaviour. The only intervention that appeared to score partially positive results across all Triple Aim domains was a care coordinator. **Conclusions:** This scoping review provides an overview of existing patient-centred interventions for multimorbidity. Findings could be used to assist in the development of a framework for multimorbid patient care. Future studies should recognise the importance of patient experience and formulate and evaluate sufficient outcomes within this domain.

## 1. Introduction

Advancements in public health and medical care have contributed to a global increase in life expectancy. The ageing and growth of the world’s population have led to an increasing number of people living with chronic conditions [1,2]. People frequently suffer from two or more long-term physical or mental conditions, referred to as multimorbidity [3,4]. A recently published systematic review mentioned prevalences of up to 93.1% among Catalan older adults (age range 65–99) [5]. While age represents a major determinant of multimorbidity, changes in lifestyle factors such as obesity and physical inactivity also play a significant role in its development. In addition, multimorbidity is associated with a certain socioeconomic status [6,7]. This growing multimorbidity trend presents significant challenges for healthcare systems, as these patients face a heightened risk of poor health outcomes. They are more likely to experience premature death, to require hospital admission and to endure longer hospital stays [8,9,10,11]. They report decreased quality of life and are more likely to suffer from mental distress [12,13,14]. Multimorbidity also imposes a substantial economic burden, with higher healthcare costs due to frequent use of medical services [8,15]. Multimorbid patients attend multiple appointments with various health professionals (including the general practitioner), increasing the risk of fragmented and ineffective care. Additionally, they are frequently prescribed complex medication regimens, leading to polypharmacy and the risk of adverse drug events and harmful drug interactions [5,16].

The increasing prevalence of multimorbidity has led to primary healthcare needs that are more complex to support. As a result, there is a need to explore alternative approaches to primary care management [17,18]. Patient-centred care (PCC) has shown promise in improving outcomes for individuals with a single chronic disease [19,20,21,22]. The US Institute of Medicine defined patient-centred care as healthcare that establishes a partnership between practitioners, patients and their families to ensure that providers and systems deliver care that is tailored to the patients’ needs, values and preferences [23]. In the growing literature, ‘patient-centredness’ is described in different ways across various studies, but they all include a set of ideas that are compatible. In this review, patient-centred care includes (1) individualised care, based on the needs and preferences of the patient, (2) from the biopsychosocial perspective, (3) in which the patient participates, (4) where the carer–patient relationship is based on shared responsibility, shared knowledge, mutual trust and empathy. (5) The communication is patient-centred, and (6) the care is coordinated in a way that maintains continuity of care [24,25].

As healthcare is currently primarily organised around single diseases, this presents unique challenges for multimorbid patients and their carers [17,26]. Research into PCC for multimorbid patients is growing, with other authors already attempting to summarise the existing literature in a systematic/scoping review [27,28,29,30,31,32]. However, the number of studies included in these reviews is often limited as there are few patient-centred intervention studies for people with multimorbidity due to methodological issues [17,28,33,34]. Such reviews seldom examine the various intervention components within the care programmes and their connection to different health outcomes [32].

The aim of this literature review was to investigate whether PCC programmes for multimorbid patients in primary care have a beneficial effect on health status, health behaviour and satisfaction. Additionally, a detailed analysis of the various intervention components was performed.

## 2. Materials and Methods

This scoping review was conducted in accordance with the methodology as described by Arksey and O’Malley [35]. They developed a stepwise approach, consisting of 5 stages, including identifying the research question, identifying relevant studies, selecting studies, presenting the data and collating, summarising and reporting on the results. The PRISMA extension for scoping reviews was followed to guide the reporting of the results (Table S1) [36]. The protocol was reported in OSF Registries, associated project osf.io/72jhf [37].

### 2.1. Information Sources

Three online databases (PubMed, Cochrane and Embase) were searched for relevant papers from November 2022. The final search was performed on 30 August 2023. A search string was developed for Pubmed, compromising the concepts ‘patient-centred care’ and ‘multimorbidity’ (Table S2). This search string was based on MeSH terms and subsequently adapted for the other databases. The grey literature, defined as ‘that which is produced on all levels of governmental, academics, business and industry in print and electronic formats, but which is not controlled by commercial publishers’ was reviewed in a second step [38]. One researcher (I.V.W.) explored the Mednar database, the WHO database, the NICE database and ClinicalTrials.gov (visited on 4 August 2023), and also performed a web search. Since it was not always possible to enter a clear search string based on index terms, we combined the keywords ‘patient-centred care’ and ‘multimorbidity’.

### 2.2. Sources of Evidence

One reviewer (I.V.W.) performed the title and abstract selection. The second reviewer was not available for this first screening. When in doubt, papers were discussed by two reviewers (I.V.W. and G.V.P.). For the full text screening, I.V.W. and G.V.P. independently evaluated the full text articles. Any disagreements were resolved during a subsequent round of discussion between I.V.W. and G.V.P. If no consensus could be reached, the opinion of two other reviewers (B.V. and N.D.) was sought. I.V.W. screened the grey literature. Any potentially interesting papers were then reviewed by both I.V.W. and G.V.P. A final discussion resulted in the list of selected articles.

### 2.3. Eligibility Criteria

Papers were eligible to be included in the review if they were published in the period from January 2000 to August 2023 and written in English or Dutch. Feasibility studies were excluded. Any papers included tested a PCC intervention, as described in the definition above. The interventions had to be performed in primary care, although collaboration with specialist care (secondary and tertiary care) or a long-term care setting were not excluded. Primary care is understood to mean a key process in the health system that supports first-contact, accessible, continued, comprehensive and coordinated patient-focused care, as defined by the WHO [39]. Study participants had to be adults (age limit defined by the country in which the study was conducted) and they had to be multimorbid as described by one of the two categories of interest:

1. Two or more chronic physical conditions. 2. At least one chronic physical condition and a cognitive or mental disorder. If these criteria were not met, the studies were not included. In each study, the various components of the definition used were examined to determine whether or not they were present.

### 2.4. Data Charting Process

A data-charting form was developed by one reviewer (I.V.W.) and discussed with a second reviewer (G.V.P.) (Table S3). One reviewer (I.V.W.) charted the data from each eligible article within both the grey and the database literature. She continuously updated the data-charting form in an iterative process. Disagreements were resolved through discussions between two reviewers (I.V.W. and G.V.P.).

The researchers extracted data on article characteristics (year of publication, country of origin), study characteristics (setting, study design, methods, eligibility criteria, age and number of participants, limitations, funding and potential conflicts of interest), intervention characteristics and outcomes.

### 2.5. Synthesis of the Results and Data Analysis

We grouped the studies by study design (Table 1) and by the type of intervention elements described (Table 2). The relationship between the intervention elements and the outcome measures was described. Outcomes were grouped based on the Triple Aim (health status, health behaviour and costs, healthcare experience of the patient and provider) [18]. Descriptive statistics were used to represent the relationship between the intervention elements and health-related outcomes of the various studies. The results are presented in Table 2. In Table S4, the various studies and their outcomes can be viewed. In the RCTs, the effect on the outcome was determined based on the odds ratios. We calculated the ratio between the number of positive outcomes and the total number of outcomes from the studies in which they were included. A colour code was assigned depending on the percentage of positive outcomes, as explained in the legend of Table 2.

## 3. Results

### 3.1. Selection of Sources of Evidence

**Figure 1:**
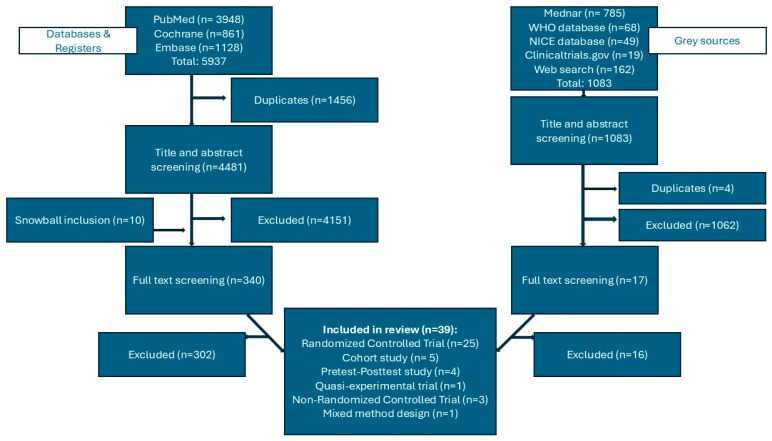
Flowchart of the screening process.

The search identified 5937 articles in databases and registers and 1083 in the grey literature (Figure 1). After removing duplicates, 5564 papers remained for title and abstract screening. Duplicates from grey sources were not removed from these results. Articles that did not meet the eligibility criteria were removed, after which 357 remained for full text screening. Of these, 318 were excluded for the following reasons: 82 did not fit the definition of multimorbidity (in several studies, patients with only one chronic disease were also included), 54 articles did not involve primary care, 68 studies included children or patients of unknown ages, 29 articles did not concern patient-centred care, 27 studies only described but did not evaluate the effect of an intervention, 38 studies involved a feasibility study, and 17 articles were not available in English or Dutch. We excluded two articles because we were not able to retrieve them.

### 3.2. Characteristics of Sources of Evidence

The studies’ place of origin, publication date, study design, characteristics of study participants, measures and outcomes are presented in Tables S4 and S5. The number of papers available per care programme varied between one and three. The papers included were mainly form the United States (*n* = 15), followed by Canada (*n* = 8), the United Kingdom (*n* = 6) and Australia (*n* = 5). The remainder originated in Puerto Rico (*n* = 1), Norway (*n* = 1), the Netherlands (*n* = 1), Spain (*n* = 1) and Chile (*n* = 1). The number of participating patients varied between 24 and 318,764 (mean 12,768) [71,77]. The most common study design was a randomised controlled trial (RCT) (*n* = 25). All interventions were performed in primary care. Some interventions (*n* = 3) involved both primary and secondary care [56,62,67]. One study was executed in diabetes education centres [54]. One care programme used a predictive model to identify multimorbid patients [41,42,43,81]. The largest number of studies included patients ≥18 years old (*n* = 16), followed by age ≥65 (*n* = 9). Some studies only mentioned the mean age (*n* = 2) or stated that the participants involved were ‘veterans’ but did not report on their exact age (*n* = 2). In these studies, it was consistently stated that the studies involved adults whereby the age limit was defined by the country where the study was conducted. One study included patients >15 years old [78]. A wide range of diseases was represented in the different papers. Some of them focused on specific conditions, while others more broadly included multiple co-existing diseases (Table S5).

In the 39 papers selected, 31 different care programmes were described. Details of the studies and the various interventions within the same programme are available in Table S5. Table 1 presents the papers and their outcomes, ordered according to the study design. Table 2 describes the intervention elements from the different care programmes in relation to the outcomes of the studies in which they were presented. The intervention elements were grouped on three levels: patient, professional and organisational. The outcomes were classified according to the Triple Aim [18].

### 3.3. Interventions

We identified 14 intervention elements described in the selected papers. These could be grouped together in three different levels according to the subdivisions made by Smith et al.: patient, professional and organisational levels (Table 2) [28].

#### 3.3.1. Patient Level

Thirty studies involved the development of an individualised and adapted care plan, the majority of which were based on goal setting (*n* = 29). Providing patient education was part of the intervention in 20 of the 27 studies focusing on self-management. Thirty-six papers addressed patient participation and shared decision making. A combination of the psychological, biological and social dimensions of care, or ‘holistic’ care, was seen in 20 papers. A large number of studies worked proactively (*n* = 33). A consideration of relatives’ needs, involving them in the patient’s care, was reported in 11 studies.

#### 3.3.2. Professional Level

In 29 studies, healthcare professionals were trained to deliver the care programme, according to the patients’ and programme’s needs. Most of these interventions focused on improving healthcare professionals’ (HCPs) general communication skills (verbal and non-verbal behaviour), characterised by an open patient-centred style (*n* = 28). A patient-centred environment was created by the HCP in 31 studies, with a focus on enhancing the patient’s motivation, understanding the patient’s situation, improving communication and focusing on a sustainable and genuine patient-–carer relationship, sharing power and responsibility.

#### 3.3.3. Organisational Level

Telemedicine was integrated in 26 studies in various ways, including using an electronic health record (EHR) with system reminders, alerts, decision support tools and audit and feedback systems, monitoring patients using home telehealth technology (apps, devices and digital patient portals), and providing telephone and video consultations. In 25 studies, a multidisciplinary team was used to deliver the care and to enhance an interdisciplinary team approach. The team’s tasks often included planning team meetings and involving a pharmacist in the daily care team. In 19 studies, a case/care manager or coordinator was appointed, who coordinated the care, the various services and the HCPs (both the team involved and the HCPs outside the care programme). The coordinator also aimed to increase communication between the patient and the healthcare system. The position was filled by a nurse, a community health worker or a trained healthcare facilitator. However, the case manager’s professional background was not specified in every article. Some studies used a ‘health coach’, who mainly provided patient empowerment or acted as a case manager [47,51,56,73]. These coaches could be medically or non-medically trained. The intervention was not led by a physician in all studies but sometimes also by another HCP or trained facilitator (Table S5) [44,45,47,48,51,53,54,56,57,62,64,65,66,73,75,77]. Some studies evaluated the patient-centred medical home model (Table 1) [69,70,71,72,75].

### 3.4. Health-Related Outcomes

As mentioned earlier, the outcomes were divided into three groups according to the care goals pursued by the Triple Aim [18]. Health status is subdivided as follows: physical health, cognitive and mental health, daily functioning, quality of life (QoL) and health-related QoL (HRQoL). The group of health behaviours includes healthcare utilisation and costs from the Triple Aim, as well as adherence to guidelines and correct prescribing behaviour by HCPs, the patient’s health behaviour (smoking behaviour, alcohol consumption, healthy eating, medication adherence and physical activity) and patient engagement, self-management and self-efficacy. The final group covers the remaining part of the Triple Aim: the healthcare experience. The outcome ‘satisfaction’ includes both patient’s and informal carer’s satisfaction with the care received, as well as the HCPs’ and trained facilitators’ satisfaction with the care model, the communication with patients and their carers, the education, the collaboration with other HCPs and the referrals. The perception of the PCC received and the quality of care (QoC), both assessed by the patient, form the final subdivisions. These outcomes were measured using various parameters, scales and questionnaires (Table S4).

### 3.5. Synthesis of the Results

As shown in Table 1, the most positive effects of the different care programmes were seen on physical health (7/11), adherence to best practices (5/7), satisfaction (5/6) and perceptions of QoC (4/5).

Patient education, patient participation, pro-active care, self-management support, patient-centred communication and a case manager were the intervention elements that were most frequently associated with a positive study outcome (a positive effect was measured in >60% of outcomes) (Table 2). Positive study outcomes of intervention elements were mainly seen on adherence to best practices, satisfaction, perceived PCC and perceived QoC.

## 4. Discussion

This review presents a comprehensive overview of patient-centred interventions in multimorbidity care, examining various intervention elements in relation to the study outcomes. It is difficult to draw firm conclusions from this research, due to the heterogeneity of both the methodology and structure of the programmes included, as well as the variability in intervention study outcomes. Nevertheless, this review provides some indications that patient-centred intervention elements may make a positive contribution to the healthcare experience of patients and carers.

Results showed that providing patient-centred approaches, such as patient participation, pro-active care, patient-centred communication and education, as well as support for patient’s self-management, are most frequently identified as having a potential positive study outcome among all the intervention elements in this review. The presence of a care coordinator or case manager was the only intervention element that scored partially positive results across all three major outcome domains (health status, health behaviour and healthcare experience). These associations do not imply causality, but our findings are largely consistent with the results that Poitras described in 2018 [32]. In this reference, only the implementation of a care coordinator had a less prominent role, while Poitras claimed the training of healthcare providers may possibly have a positive impact on the care of multimorbid patients [32]. Comparing the literature, Smith indicated that case management had mixed effects, with improvements in client and professional satisfaction with care and reductions in carer strain, but no impact on healthcare utilisation [28]. Sadler reported that the evidence was uncertain regarding case management for frail older people [82].

In our review, the PCC programmes proved to have possible beneficial effects on adherence to best practices, satisfaction and perceptions of QoC. Similar to our findings, de Bruin et al. cited moderate evidence for a beneficial effect of patient-centred comprehensive care programmes on patients’ health behaviour, perceived quality of care and patients’ and carers’ satisfaction. Insufficient or no evidence was found regarding a beneficial effect on health-related quality of life, cognitive and mental health, medication use, and outpatient healthcare utilisation and healthcare costs [31]. Smith et al. also described limited effects on HRQoL and outcomes related to healthcare utilisation. Improvements in outcomes were observed among organisational interventions [28]. Sogaard, on the other hand, reported that patient engagement interventions had a positive effect on health status, HRQoL and healthcare utilisation [29].

Almost all of our intervention elements were associated with a (partially) positive study outcome on healthcare experience, while only a portion of the interventions showed positive study outcomes for health behaviour and almost none for health status. However, maximising the impact of PCC programmes and intervention elements on the other two axes of the Triple Aim (and also on the Quadruple Aim) remains a challenge. Although the most beneficial effects are seen in patient experience, most studies focus on other outcomes (health status and health behaviour). It is important for future studies to recognise the importance of patient experience and to formulate and evaluate sufficient outcomes related to the field of healthcare experience [83].

However, we must take into account that patient-centred interventions are complex, as well as the fact that they are difficult to measure. As previously mentioned, patient-centred care is described in varying ways across studies. In the research, patient participation, patient engagement, self-management and PCC are sometimes used interchangeably, although these are three separate concepts. Patient empowerment is broader than patient participation and patient-centredness, while patient participation facilitates a patient-centred approach [25]. In addition, the content of the various intervention elements is very diverse. In some studies, the case manager or care coordinator fulfils an exclusively administrative role, while in other studies, they also have a clinical function. Studies used a variety of outcome measures in all outcome domains (Table S4). There was also a large variability in the number of participants between the different studies. This made it challenging to pool and interpret the results.

The definition of the concept of multimorbidity also posed several challenges. The first problem was the absence of a clear definition of multimorbidity, as discussed earlier in this article. Many studies were not included in this review because they did not meet our definition criteria. However, one must take into account that a different definition could possibly lead to a different synthesis and conclusion. Furthermore, where studies did appear to meet the definition, we struggled with the interpretation of several aspects, including the question of which elements in people’s health should be considered chronic diseases. For instance, some studies considered hypertension a chronic disease, whereas other studies considered this to be a risk factor. On this topic, Ho et al. developed a list of conditions that always must be included in multimorbidity measures [3]. Ho’s list was helpful in determining whether certain conditions should be viewed as a chronic disease or a risk factor. Finally, some studies used an incorrect definition or confused multimorbidity with another concept. For example, multimorbidity was used interchangeably with frailty in some papers. Although multimorbidity and frailty are highly associated, they are not the same [84,85]. A systematic review of Vetrano et al. revealed that 72% of frail individuals present multimorbidity, but only 16% of individuals with multimorbidity are also frail [84].

In addition, we noticed that patients with mental or cognitive illness were excluded from several studies. This certainly deserves special attention given the high prevalence of these conditions among multimorbid patients [9]. The reason why these patient groups were excluded was not stated, but it is possibly due to the difficulty of research in this group. Often, only the elderly were included in the multimorbidity research (Table S5). However, multimorbidity, as mentioned before, is not solely driven by age. In absolute numbers, more people < 65 years of age are multimorbid than people ≥ 65 years of age, partly because there are more people belonging to this age group [7,50]. These findings all contribute to the complexity of multimorbidity research. Nevertheless, this review provides interesting insights into the different intervention elements in PCC programmes.

By presenting the different intervention elements and their associations with study outcomes, this review has the potential to serve as a foundation for developing a theoretical framework applicable to multimorbidity care, tailored to individual patient needs. This framework can serve as a reference for evaluating current clinical practice, facilitating the identification of existing gaps. In this way, a gradual transition from disease-centred to patient-centred care could be achieved.

This review provided an additional look at previously published work on PCC for multimorbid patients. A new aspect covered in this work is the different intervention elements that were examined. Both databases and the grey literature were searched. The review included studies that did not focus exclusively on elderly populations. In addition, cognitive and mental illness were also covered in the definition of multimorbidity that was used in this research. We included both studies with positive and negative outcomes.

Despite the strengths, some limitations should be noted. Most of the studies included were conducted in countries with a well-developed healthcare system. These results may not be representative of other healthcare systems. In addition, some studies only had a small number of participants. Follow-up periods for the interventions were usually short-term, ranging from several months to one year. There was heterogeneity in outcome measurement. An additional limitation includes potential screening bias due to partial single-reviewer extraction. Finally, we must emphasise that we attempted to study all the various elements of patient-centred interventions. However, the reader must always keep in mind that these types of studies are complex and often involve multiple intervention elements in one care programme. It is therefore impossible to conclude a clear relationship between the elements and the outcomes, since the outcome may be the result of a combination of several elements. The isolated use of one intervention element in another study is therefore no guarantee of success.

## 5. Conclusions

Given the importance of developing interventions for people with multimorbidity, this review provides interesting insights into the different intervention elements in PCC programmes. Evidence suggests potential benefits primarily for patient experience outcomes. Future studies should recognise the importance of patient experience and also formulate sufficient outcomes in this regard.

## Figures and Tables

**Table 1:** Overview of the studies included and their outcomes. Green: Significant positive effect of the study on this outcome. Blue: Positive, but no significant effect. Orange: No change of the outcome compared with the control group. Red: Negative effect of the study on this outcome. (HR)QoL: (health-related) quality of life. PE: patient engagement. SM: self-management. SE: self-efficacy. PCC: patient-centred care. QoC: quality of care. PACT*: patient-centred team. PACT**: patient-aligned care team initiative.

Study Design	Authors	Year	Programme	Outcomes																							
				Health Status							Health Behaviour													Healthcare Experience			
				Physical Health		Functioning	Cognitive & Mental Health		(HR)QoL		Healthcare Utilisation			Healthcare Costs	Adherence to Best Practices			Healthy Behaviours			PE/SM/SE			Satisfaction		Perceived PCC	Perceived QoC
RCT	Blom [40]	2016	ISCOPE																								
RCT	Boult, Boyd, Wolff [41,42,43]	2008, 2009, 2010	Guided Care																								
RCT	Coventry, Camacho [44,45]	2015, 2016	COINCIDE																								
RCT	del Cura-González [46]	2022	MULTIPAP																								
RCT	Contant [47]	2019	PR1MaC Trial																								
RCT	Fisher [48]	2020																									
RCT	Fortin, Ryan [49,50]	2021, 2023	IMPACT plus																								
RCT	Hochhalter [51]	2010	Making the Most of Your Healthcare																								
RCT	Katon [52]	2010																									
RCT	Khunti [53]	2021	MAP																								
RCT	Markle-Reid [54]	2018																									
RCT	Morgan [55]	2012	TrueBlue																								
RCT	Naik [56]	2019	HOPE																								
RCT	Reed [57]	2018	CDSMS																								
RCT	Salisbury, Thorn [58,59]	2018, 2020	The 3D Approach																								
RCT	Miranda [60]	2022	ePRO tool																								
RCT	Stewart [61]	2021	Telemedicine IMPACT Plus																								
RCT	Vasan [62]	2020	IMPaCT																								
RCT	Vera [63]	2010																									
RCT	Wakefield [64]	2011																									
Intervention-only design	Abadi [65]	2022	TCMLH																								
Pretest-post-test study	Ansari [66]	2020	APCOM																								
Prospective propensity score matched controlled trial	Berntsen [67]	2019	PACT*																								
Pretest-post-test study	Fortin [68]	2021	IMPACT plus																								
Pretest-post-test study, controlled cohort study	John, John [69,70]	2020, 2020	WellNet																								
Cohort study	Schuttner, Schuttner [71,72]	2019, 2020	PACT**																								
Quasi-experimental design	Shah [73]	2019	Enhanced Primary Care Model																								
Controlled cohort study	Sommers [74]	2000	SCC																								
Non-RCT	Swietek [75]	2018	CCNC																								
Non-RCT	Tinetti [76]	2019																									
Pretest-post-test study	Yamane [77]	2020																									
Cohort study	Zamorano [78]	2022	MPCM																								
(Partially) positive outcome in				7/11		1/4	9/18		10/22		7/13			1/7	5/7			5/13			1/2			5/6		2/5	4/5

**Table 2:** Intervention elements and the outcomes of the studies in which they were included. This table shows the different intervention elements and the ratio between the number of positive outcomes and the total number of outcomes from the studies in which they were included. A colour code was assigned depending on the percentage of positive outcomes.

Level	Intervention Elements	Study Outcomes											
		Health Status				Health Behaviour					Healthcare Experience		
		Physical Health	Functioning	Cognitive & Mental Health	(HR)QoL	Healthcare Utilisation	Healthcare Costs	Adherence to Best Practices	Healthy Behaviours	PE/SM/SE	Satisfaction	Perceived PCC	Perceived QoC
Patient	Care plan [40,41,42,43,44,45,46,47,48,49,52,54,55,56,57,58,59,60,61,62,65,66,67,68,69,70,74,77,78,79,80]	3/7	0/2	10/23	13/43	21/46	1/10	5/9	5/23	14/27	6/11	8/9	1
	Goal setting [40,41,42,43,44,45,46,47,48,49,52,55,56,57,58,59,60,61,62,65,66,67,68,69,70,74,76,78,79,80]	3/7	0/2	3/7	13/43	2/5	1/9	5/9	2/11	12/25	6/11	7/9	4/5
	Holistic care [40,44,45,46,47,48,52,54,55,56,57,58,59,62,65,69,70,71,72,73,75,78]	3/8	0/2	4/9	4/11	1/2	1/7	11/17	1/10	1/2	10/19	7/10	1
	Patient education [41,42,43,44,45,47,52,53,56,63,64,65,66,69,70,73,74,75,77,78]	9/26	1/3	7/13	4/9	5/8	1	11/14	2/11	12/19	1	1/3	1
	Patient participation [40,41,42,43,44,45,46,47,48,49,51,52,54,55,56,57,58,59,60,61,62,63,64,65,67,68,69,70,71,72,73,74,75,76,77,78,79,80]	15/33	1/4	13/30	14/47	23/51	1/10	13/19	1/5	3/7	6/11	7/11	5/6
	Pro-active care [40,41,42,43,44,45,47,48,49,52,53,54,55,56,57,58,59,60,61,62,64,65,66,67,68,69,70,71,72,74,75,77,78,80]	5/16	0/2	12/29	14/45	21/43	1/10	11/17	3/14	14/29	6/11	1	1
	Self-management support [41,42,43,45,47,48,49,52,53,54,55,56,57,60,64,65,66,68,69,70,71,72,73,74,77,78,79,80]	16/47	0/2	11/26	14/35	13/28	1/6	4/5	1/4	1/2	1	1/3	1
	Informal carer involvement [40,41,42,43,48,53,54,60,62,67,74]	1/9	0/2	1/10	1/10	5/9	0/5		1/6	1/6	4/13		1
Professional	Training healthcare providers [40,41,42,43,44,45,46,47,48,49,52,54,55,56,58,59,62,63,65,66,68,71,72,73,74,76,77,78,79]	3/7	1/4	6/13	11/36	20/43	1/8	3/5	5/18	12/23	4/7	7/11	5/6
	Patient-centred communication [41,42,43,44,45,46,47,48,49,52,54,55,58,59,62,65,66,67,68,69,70,71,72,73,76,77,78]	4/9	0/1	10/21	2/5	20/39	1/7	2/3	5/17	14/25	1	7/11	5/6
	Patient-centred environment [41,42,43,44,45,46,47,48,49,52,54,55,56,57,58,59,61,62,65,66,67,68,69,70,71,72,73,74,76,77,78,79,80]	13/31	0/2	4/9	15/43	23/51	1/7	3/5	5/24	14/29	10/11	7/11	5/6
Organisational	Telemedicine and decision support [41,42,43,44,45,48,49,51,52,56,57,58,59,60,61,63,64,68,69,70,71,72,73,74,76,77,78]	13/22	1/3	11/24	1/4	13/29	1/7	5/9	2/17	6/17	6/7	7/10	5/6
	Care coordinator [41,42,43,44,45,48,54,55,62,63,64,67,69,70,71,72,75,76,78,80]	9/23	1	1/2	2/5	17/26	1/4	4/5	1/8	4/11	3/4	0/1	4/5
	Multidisciplinary team [40,41,42,43,44,45,48,49,52,54,55,58,59,63,67,68,69,70,78,79,80]	13/28	1/3	6/13	9/34	1/2	1/8	4/7	1/5	2/5	4/9	6/7	1
0–20%	(HR)QoL: (health-related) quality of life. PE: patient engagement. SM: self-management. SE: self-efficacy. PCC: patient-Ccentred care. QoC: quality of care.												
21–40%													
41–60%													
61–80%													
81–100%													

## Data Availability

The original contributions presented in this study are included in the article/Supplementary Material. Further inquiries can be directed to the corresponding author.

## Supplementary Materials

The following supporting information can be downloaded at: https://www.mdpi.com/article/10.3390/jcm15103774/s1, Table S1: PRISMA-ScR Checklist; Table S2: Search strategy; Table S3: Data-charting form; Table S4: Overview of the studies included and their outcomes; Table S5: Overview of the study characteristics.

## Abbreviations

The following abbreviations are used in this manuscript:

PCC	Patient-centred care
HCP	Healthcare professional
EHR	Electronic health record
QoL	Quality of life
HRQoL	Health-related quality of life
PE	Patient engagement
SM	Self-management
SE	Self-efficacy
QoC	Quality of care
