# Supplementary material for: Patient-Centred Care for Multimorbid Patients: A Scoping Review
Source: J Clin Med. 2026 May 14;15(10):3774. doi: 10.3390/jcm15103774 (PMC13207952; doi:10.3390/jcm15103774)
Supplement: Supplementary file 1 [file jcm-15-03774-s001.zip › Table_S2.pdf]

Table S2: Search strategy.

PubMed/Medline

Search string for 'Patient centered care'
"patient centered care"[MeSH Terms] OR "patient centered care"[MeSH Terms] OR ("patient centered"[All Fields] AND "care"[All Fields]) OR "patient centered care"[All Fields] OR ("patient"[All Fields] AND "centered"[All Fields] AND "care"[All Fields]) OR "patient centered care"[All Fields] OR "patient centered care"[MeSH Terms] OR ("patient centered"[All Fields] AND "care"[All Fields]) OR "patient centered care"[All Fields] OR ("person"[All Fields] AND "centered"[All Fields] AND "care"[All Fields]) OR "person centered care"[All Fields] OR ("patient s"[All Fields] OR "patients"[MeSH Terms] OR "patients"[All Fields] OR "patient"[All Fields] OR "patients s"[All Fields]) AND "centred"[All Fields] AND "care"[All Fields]) OR ("person s"[All Fields] OR "personable"[All Fields] OR "personableness"[All Fields] OR "personal"[All Fields] OR "personalisation"[All Fields] OR "personalise"[All Fields] OR "personalised"[All Fields] OR "personalising"[All Fields] OR "personality"[MeSH Terms] OR "personality"[All Fields] OR "personalities"[All Fields] OR "personality s"[All Fields] OR "personalization"[All Fields] OR "personalize"[All Fields] OR "personalized"[All Fields] OR "personalizes"[All Fields] OR "personalizing"[All Fields] OR "personally"[All Fields] OR "personals"[All Fields] OR "persons"[MeSH Terms] OR "persons"[All Fields] OR "person"[All Fields]) AND "centred"[All Fields] AND "care"[All Fields]
**AND**
Search string for 'Multimorbidity'
"chronic disease"[MeSH Terms] OR "comorbidity"[MeSH Terms] OR ("multimorbid"[All Fields] OR "multimorbidities"[All Fields] OR "multimorbidity"[MeSH Terms] OR "multimorbidity"[All Fields]) OR ("multimorbid"[All Fields] OR "multimorbidities"[All Fields] OR "multimorbidity"[MeSH Terms] OR "multimorbidity"[All Fields]) OR "multi-morbidity"[All Fields] OR "multi-morbidities"[All Fields] OR ("multiple chronic conditions"[MeSH Terms] OR ("multiple"[All Fields] AND "chronic"[All Fields] AND "conditions"[All Fields]) OR "multiple chronic conditions"[All Fields]

Embase

('multiple chronic conditions'/exp OR 'multiple chronic conditions' OR 'comorbidity'/exp OR 'comorbidity') AND ('person centered care'/exp OR 'person centered care')

Cochrane

Search string for 'Patient centered care'
[patient adj centered adj care OR patient adj centred adj care OR person adj centered adj care OR person adj centred adj care OR care, patient centered[MeSH Terms]]
**AND**
Search string for 'Multimorbidity'
[Multimorbidity OR Multi-morbidity OR multimorbidities OR multi-morbidities OR Multiple chronic conditions (MeSH) OR chronic disease (MeSH) OR comorbidity (MeSH)]
