# Supplementary material for: Patient-Centred Care for Multimorbid Patients: A Scoping Review
Source: J Clin Med. 2026 May 14;15(10):3774. doi: 10.3390/jcm15103774 (PMC13207952; doi:10.3390/jcm15103774)
Supplement: Supplementary file 1 [file jcm-15-03774-s001.zip › Table_S3.pdf]

Table S3: Data charting form.

Authors	Title	Year	Location	Definition multimorbidity	Setting Primary care?	Study design	Study methods	Exclusion criteria	Type of PCC Intervention	Program characteristics	Age	Number of participants	Data analysis	Outcome			Results	Program	Limitations	Funding source, conflicts of interest
														Health status	Health behavior	Satisfaction				
