# Supplementary material for: Patient-Centred Care for Multimorbid Patients: A Scoping Review
Source: J Clin Med. 2026 May 14;15(10):3774. doi: 10.3390/jcm15103774 (PMC13207952; doi:10.3390/jcm15103774)
Supplement: Supplementary file 1 [file jcm-15-03774-s001.zip › Table_S4.pdf]

Table 4: Overview of the included studies and their outcomes.

	Authors	Outcomes												
		Health status					Health behaviour					Healthcare experience		
		Physical health	Functioning	Cognitive & mental health	QoL	HRQoL	Healthcare utilization	Healthcare costs	Adherence to best practices	Healthy behaviours	PE/SM/SE	Satisfaction	Perceived PCC	Perceived QoC
1.	Abadi					PROMIS-10: mental health + *physical health* , *PSS-4* , Meaning and Purpose Scale					*Patient motivation measure* , Health Care Empowerment Questionnaire, The goal-specific hope scale: *pathways* + *agency*, Whole Health self-care practices, Goal progress	Program and facilitator satisfaction, attitudes about group programs, attitudes about Whole Health self-care practices		
2.	Ansari					*CAT*, MULTIPleS, COPD MULTIPLes					*PAM13*, *COPD-Q* , *Proper inhaler device technique*			
3.	Berntsen	All cause mortality					Emergency admissions, the sum of emergency inpatient bed days, 30-day readmissions, planned outpatient visits, emergency outpatient visits							
4.	Blom		GARS	GDS-15, MMSE	Cantril's ladder	DJG		QALY				**Patients:** satisfaction with and confidence in their GP, PH, S, PT, hospital and home care. **GPs:** overview of care needs, stability in the care situation and improvement in the care situation, experiences of the GPs with the screening and care plans. **Informal caregivers:** hours per week spent on household activities, personal care and activities outside the house, the burden of this care and their quality of life		
5.	Boult											**Satisfaction by the GP:** communication with patients, communication with family caregivers, education of caregivers, motivating patients to participate in their care, referrals to community resources and knowing all medications that patients are taking. **Satisfaction by the N:** a validated nurses' job satisfaction instrument		PACIC
6.	Boyd													PACIC
7.	Wolff			CESD-10, CSI							Work Productivity and Activity Impairment questionnaire			modified PACIC
8.	Camacho			SCL-D13				QALY						
9.	Coventry			SCL-D13, PHQ-9, GAD-7, SDS	WHOQOL-BREF	MULTIPLeS, Diabetes quality of life and Seattle angina questionnaire					HeiQ, self efficacy	CSQ		PACIC

10.	del Cura-González	Incidence of drug adverse events				EQ-5D-5L	Hospitalizations, visits to emergency services, and number of FP and primary care nurse visits		MAI	Morisky Medication Adherence score			collaboRATE	
11.	Contant										HeiQ			
12.	Fisher			CESD-10, GAD-7		SF-12: PCS, MCS	HSSUI	Total service costs			Self-Efficacy for Managing Chronic Disease Scale			
13.	Fortin			K6		VR-12: PCS and MCS, EQ-5D-5L				Smoking, alcohol, *healthy eating*, physical activity	*HeiQ*, SE-CD			
14.	Fortin			K6		VR-12: PCS and MCS, EQ-5D-5L				Smoking, alcohol, *healthy eating*, physical activity	HeiQ, SE-CD			
15.	Ryan						Acute hospital admissions, emergency department visits, 30-day hospital readmissions, 7-day follow-up with GP	Total costs						
16.	Hochhalter					CDC Healthy Days Measure, HRQOL-14					PAM, Self-Efficacy for Managing Chronic Disease assessment			
17.	John					EQ-5D-5L, KOOS, HOOS					PAM13			
18.	John										PAM13			
19.	Katon	HbA1c, LDL, SBP		SCL-20	Quality of life over the previous 1-month period on a scale from 0 to 10				Adjustments of insulin, antihypertensive medications, antidepressant medications	Days of adherence to diet and exercise		Satisfaction with care for depression and for DM, CHD		
20.	Khunti	TC, LDL, HDL, TG, kidney function, HbA1c, BMI, BW, waist circumference, hip circumference, waist/hip ratio, left-hand grip, right-hand grip, SBP, DBP, HR		HADS		EQ-5D-5L				Recent Physical Activity Questionnaire, ASK-12, change in overall volume of daily physical activity, dietary behaviour, sleeping pattern	Chronic Disease Self-Efficacy Scale, SEE			
21.	Markle-Reid			GAD-7, CES-D-10		SF-12: PCS, MCS		Healthcare costs			SDSCA, Self-Efficacy for Managing Chronic Disease.			
22.	Morgan	BMI, waist circumference, SBP, TC, LDL, HDL, TG, HbA1C, 10-year CVD risk in T2DM		PHQ-9 depression score		SF-36: PCS, MCS	Referrals to exercise programmes, attendsto exercise program, referrals to PWP and visits to PWP			Smoking, alcohol, excercises 30min/day 5days/week				
23.	Naik	HbA1c		*PHQ-9 depression score*			Mental health clinic visits, primary care visits or diabetes medication prescriptions		Increase, decrease, or stop of medications					
24.	Reed	Fatigue, Pain		Energy, Depression		Self-rated health on a 5-point Likert scale, health distress, illnessintrusiveness	GP visits, emergency department visits, hospital admissions			Exercise, medication adherence	Self-efficacy, heiQ			
25.	Salisbury				The Hospital Anxiety and Depression score	EQ-5D-5L, Bayliss measure, self-rated health, treatment burden	The number of different drugs prescribed, hospital admissions and outpatient attendances, nurse consultations and GP visits		High-risk prescribing, The proportion of UK Quality and Outcomes Framework chronic disease targets applicable to each patient that were met	Morisky Medication Adherence score			PACIC, CARE measure of relational empathy, and single questions about the proportion of patients reporting care related to their priorities, those experiencing their care as joined up, those reporting having a written care plan, and overall satisfaction with care, COCI and the Visit Entropy measure	
26.	Thorn							QALY, costs, ICER						
27.	Schuttner					SF-12: PCS and MCS								

28.	Schuttner	**CHF patients:** **LVEF < 40%, on RAAS-inhibition.** **DM patients**: LDL <100, **BP <140/90 mmHg,** HA1C < 9%, **Foot exam.** **IHD patient:** LDL < 100 **Vaccinated patients:** **18–64 years flu, > 6 years flu,** >65y flu, **> 65 years PPSV23.**		PTSD screening											
29.	Shah		**Physical functioning**	**Anxiety, depression**		**EQ-5D-5L**	Time since last GP appointment, time since last nurse appointment			**Smoking habit**	**Resilience**		**Rating of GP person-centeredness, frequency of seeing preferred GP (continuity)**	
30.	Sommers	*Symptom scale*	HAC	GDS		*SF-36*	*Hospital admissions per patient , readmissions , mean number of office visits to all physicians,* emergency department visits, proportion of patients with 1 or more skilled nursing facility admission, proportion of patients with 1 or more home care visits, medications count.			Nutrition check-list				
31.	Swietek								**9 disease specific quality indicators to evaluate the receiving of recommended health services:** HA1C testing, attention for nephropathy, eye examinations, liver function tests, lipid profile testing for persons with DM and/or hyperlipidemia, receipt of RAAS-inhibition, **SABA overuse,** psychotherapy for persons with major depression and persons with schizophrenia, ACT for schizophrenia					
32.	Miranda					AQoL-4D		**QALY, costs**						
33.	Stewart			K6, *Mental health status*		**SF-12:** PCS and MCS, EQ-5D-5L				**Health behaviour survey:** no alcohol, physical activity >= 2/week, healthy eating, **healthy BMI**	HeiQ, SEM2	Patients' experiences of the intervention		
34.	Tinetti					TBQ	Odds of receiving diagnostic tests, **procedures scheduled or** avoided, **GP visits** avoided **and ordered**		Medications stopped		**Self-management tasks added and stopped**		CollaboRATE	O-PACIC
35.	Vasan						All-cause hospitalization, the proportion of hospitalizations occurring outside a patient's primary health system, average length of stay, % of patients with multiple hospitalizations, 30-day readmission rate, DRG weight							
36.	Vera			SCL-20		SF-36								
37.	Wakefield	HbA1c, *SBP*								Medication adherence				
38.	Yamane								Provider adherence to prescription guidelines for the management of HT	Medication adherence	High BP quiz		Communication between provider and patient	
39.	Zamorano	All cause mortality					Hospital admissions, length of stay, the number of hospital emergency consultancies, number of consultancies to primary care emergency, number of consultancies to specialists at hospital, number of consultations to GP							

Green = Significant positive effect of the study on this outcome. Blue = Positive, but no significant effect. Orange = No change of the outcome towards the control group. Red = Negative effect of the study on this outcome. (HR)QoL = Health Related Quality of Life. PE = Patient engagement. SM = Self- management. SE = Self-efficacy. PCC = Patient-Centered Care. QoC = Quality of Care. HbA1C = Glycated haemoglobin. LDL = Low density lipoprotein. TG = Triglycerides. TC = Total cholesterol. (S)BP = (Systolic) blood pressure. HT = Hypertension. BW = Body weight. HR = Heart rate. CHF = Chronic heart failure. LVEF= Left ventricular ejection fraction. DM = Diabetes mellitus. IHD = Ischemic heart disease. PPSV23 = Pneumococcal polysaccharide vaccine. CESD-10 = Center for Epidemiologic Studies Depression Scale. CSI = Modified Caregiver Strain Index. SDS = Sheehan disability scale. GAD-7 = Generalised anxiety disorder-7. K6 = Kessler 6-item Psychological Distress Scale Questionnaire. HADS = Hospital anxiety and depression scale. HAC = Health Activities Questionnaire. GDS = Geriatric depression scale. SF-36 = 36-Item Short-Form Health Survey. GARS = Groningen Activities Restriction Scale. WHOQOL-BREF = World Health Organization Quality of Life Brief Version. CAT = COPD assessment test. MULTIPleS = Multimorbidity Illness Perceptions Scale. COPD MULTIPLes = COPD related multi-morbidity. DJG = Loneliness Scale of De Jong Gierveld. SF-12= 12-Item Short-Form health survey. PCS = Physical component summary. MCS = Mental component summary. VR-12 = Veterans RAND 12 Item Health Survey. KOOS = Knee Injury and Osteoarthritis Outcome Score. HOOS = Hip Disability and Osteoarthritis Outcome Score. AQoL-4D = Assessment of Quality of Life 4-Dimension. TBQ = Treatment Burden Questionnaire. MAI = Medication Appropriateness Index. HSSUI = Health and Social Services Utilization Inventory. DRG = Diagnosis-related group. QALY = Cost per adjusted life year, estimated from the EQ -5D-5L and utility tariffs. ICER = Incremental cost-effectiveness ratio. ACT = Assertive community treatment. ASK-12 = Adherence Starts with Knowledge questionnaire. PAM13 = Patient Activation Measure 13. COPD-Q = The COPD Knowledge Questionnaire. HeiQ = Health education impact questionnaire. SE-CD = Self-Efficacy for Managing Chronic Diseases. SEE = Self-Efficacy for Exercise scale. SDSCA = Summary of Diabetes Self-Care Activities. SEM2 = Self-Efficacy for Managing Chronic Disease scale. PACIC = Patient Assessment of Chronic Illness Care. CSQ = The client satisfaction questionnaire. CARE = The Consultation and Relational Empathy. O-PACIC = Older Patient Assessment of Chronic Illness Care. COCI = Continuity of Care index.

References

1. Abadi, M.; Richard, B.; Shamblen, S.; Drake, C.; Schweinhart, A.; Bokhour, B.; et al. Achieving Whole Health: A Preliminary Study of TCMLH, a Group-Based Program Promoting Self-Care and Empowerment Among Veterans. *Health Educ Behav* **2022,** *49*, 347-57. doi: 10.1177/10901981211011043.
2. Ansari, S.; Hosseinzadeh, H.; Dennis, S.; Zwar, N. Activating primary care COPD patients with multi-morbidity through tailored self-management support. *NPJ Prim Care Respir Med* **2020,** *30*, 12. doi: 10.1038/s41533-020-0171-5.
3. Berntsen, G.K.R.; Dalbakk, M.; Hurley, J.S.; Bergmo, T.; Solbakken, B.; Spansvoll, L.; et al. Person-centred, integrated and pro-active care for multi-morbid elderly with advanced care needs: a propensity score-matched controlled trial. *BMC Health Serv Res* **2019,** *19*, 682. doi: 10.1186/s12913-019-4397-2.
4. Blom, J.; den Elzen, W.; van Houwelingen, A.; Heijmans, M.; Stijnen, T.; Van den Hout, W.; et al. Effectiveness and cost-effectiveness of a proactive, goal-oriented, integrated care model in general practice for older people. A cluster randomised controlled trial: Integrated Systematic Care for older People-the ISCOPE study. *Age Ageing* **2016,** *45*, 30-41. doi: 10.1093/ageing/afv174.
5. Boult, C.; Reider, L.; Frey, K.; Leff, B.; Boyd, C.; Wolff, J.; et al. Early effects of "Guided Care" on the quality of health care for multimorbid older persons: a cluster-randomized controlled trial. *J Gerontol A Biol Sci Med Sci* **2008,** *63*, 321-7. doi: 10.1093/gerona/63.3.321.
6. Boyd, C.; Reider, L.; Frey, K.; Scharfstein, D.; Leff, B.; Wolff, J.; et al. The Effects of Guided Care on the Perceived Quality of Health Care for Multi-morbid Older Persons: 18-Month Outcomes from a Cluster-Randomized Controlled Trial. *J Gen Intern Med* **2010,** *25*, 235-42. doi: 10.1007/s11606-009-1192-5.
7. Wolff, J.L.; Giovannetti, E.R.; Boyd, C.M.; Reider, L.; Palmer, S.; Scharfstein, D.; et al. Effects of guided care on family caregivers. *Gerontologist* **2010,** *50*, 459-70. doi: 10.1093/geront/gnp124.
8. Camacho, E.M.; Ntais, D.; Coventry, P.; Bower, P.; Lovell, K.; Chew-Graham, C.; et al. Long-term cost-effectiveness of collaborative care (vs usual care) for people with depression and comorbid diabetes or cardiovascular disease: a Markov model informed by the COINCIDE randomised controlled trial. *BMJ open* **2016,** *6*, e012514. doi: 10.1136/bmjopen-2016-012514.
9. Coventry, P.; Lovell, K.; Dickens, C.; Bower, P.; Chew-Graham, C.; McElvenny, D.; et al. Integrated primary care for patients with mental and physical multimorbidity: cluster randomised controlled trial of collaborative care for patients with depression comorbid with diabetes or cardiovascular disease. *BMJ* **2015,** *350,* h638. doi: 10.1136/bmj.h638.
10. Del Cura-González, I.; López-Rodríguez, J.; Leiva-Fernández, F.; Gimeno-Miguel, A.; Poblador-Plou, B.; López-Verde, F.; et al. How to Improve Healthcare for Patients with Multimorbidity and Polypharmacy in Primary Care: A Pragmatic Cluster-Randomized Clinical Trial of the MULTIPAP Intervention. *J Pers Med* **2022,** *12*, 752. doi: 10.3390/jpm12050752.
11. Contant, É.; Loignon, C.; Bouhali, T.; Almirall, J.; Fortin, M. A multidisciplinary self-management intervention among patients with multimorbidity and the impact of socioeconomic factors on results. *BMC Fam Pract* **2019,** *20*, 53. doi: 10.1186/s12875-019-0943-6.
12. Fisher, K.; Markle-Reid, M.; Ploeg, J.; Bartholomew, A.; Griffith, L.E.; Gafni, A.; et al. Self-management program versus usual care for community-dwelling older adults with multimorbidity: A pragmatic randomized controlled trial in Ontario, Canada. *J Comorb* **2020,** *10,* 2235042X20963390. doi: 10.1177/2235042X20963390.
13. Fortin, M.; Stewart, M.; Almirall, J.; Berbiche, D.; Bélanger, M.; Katz, A.; et al. One year follow-up and exploratory analysis of a patient-centered interdisciplinary care intervention for multimorbidity. *J Multimorb Comorb* **2021,** *11,* 26335565211039780. doi: 10.1177/26335565211039780.

14. Fortin, M.; Stewart, M.; Ngangue, P.; Almirall, J.; Bélanger, M.; Brown, J.B.; et al. Scaling Up Patient-Centered Interdisciplinary Care for Multimorbidity: A Pragmatic Mixed-Methods Randomized Controlled Trial. *Ann Fam Med* **2021,** *19*,126-34. doi: 10.1370/afm.2650.
15. Ryan, B.L.; Mondor, L.; Wodchis, W.P.; Glazier, R.H.; Meredith, L.; Fortin, M.; et al. Effect of a multimorbidity intervention on health care utilization and costs in Ontario: randomized controlled trial and propensity-matched analyses. *CMAJ Open* **2023,** *11*, e45-e53. doi: 10.9778/cmajo.20220006.
16. Hochhalter, A.K.; Song, J.; Rush, J.; Sklar, L.; Stevens, A. Making the Most of Your Healthcare intervention for older adults with multiple chronic illnesses. *Patient Educ Couns* **2010,** *81*, 207-13. doi: 10.1016/j.pec.2010.01.018.
17. John, J.R.; Tannous, W.K.; Jones, A. Outcomes of a 12-month patient-centred medical home model in improving patient activation and self-management behaviours among primary care patients presenting with chronic diseases in Sydney, Australia: a before-and-after study. *BMC Fam Pract* **2020,** *21*, 158. doi: 10.1186/s12875-020-01230-w.
18. John, J.R.; Jones, A.; Neville, A.M.; Ghassempour, S.; Girosi, F.; Tannous, W.K. Cohort Profile: Effectiveness of a 12-Month Patient-Centred Medical Home Model Versus Standard Care for Chronic Disease Management among Primary Care Patients in Sydney, Australia. *Int J Environ Res Public Health* **2020,** *17*, 2164. doi: 10.3390/ijerph17062164.
19. Katon, W.J.; Lin, E.H.; Von Korff, M.; Ciechanowski, P.; Ludman, E.J.; Young, B.; et al. Collaborative care for patients with depression and chronic illnesses. *N Engl J Med* **2010,** *363*, 2611-20. doi: 10.1056/NEJMoa1003955.
20. Khunti, K.; Highton, P.J.; Waheed, G.; Dallosso, H.; Redman, E.; Batt, M.E.; et al. Promoting physical activity with self-management support for those with multimorbidity: a randomised controlled trial. *Br J Gen Pract* **2021,** *71*, e921-e30. doi: 10.3399/BJGP.2021.0172.
21. Markle-Reid, M.; Ploeg, J.; Fraser, K.; Fisher, K.; Bartholomew, A.; Griffith, L.; et al. Community Program Improves Quality of Life and Self-Management in Older Adults with Diabetes Mellitus and Comorbidity. *J Am Geriatr Soc* **2018,** *66*, 263-273. doi: 10.1111/jgs.15173.
22. Morgan, M.A.; Coates, M.J.; Dunbar, J.A.; Reddy, P.; Schlicht, K.; Fuller, J. The TrueBlue model of collaborative care using practice nurses as case managers for depression alongside diabetes or heart disease: a randomised trial. *BMJ Open* **2013,** *3*, e002171. doi: 10.1136/bmjopen-2012-002171.
23. Naik, A.; Hundt, N.; Vaughan, E.; Petersen, N.; Zeno, D.; Kunik, M.; et al. Effect of Telephone-Delivered Collaborative Goal Setting and Behavioral Activation vs Enhanced Usual Care for Depression Among Adults With Uncontrolled Diabetes: A Randomized Clinical Trial. *JAMA Netw Open* **2019,** *2*, e198634. doi: 10.1001/jamanetworkopen.2019.8634.
24. Reed, R.L.; Roeger, L.; Howard, S.; Oliver-Baxter, J.M.; Battersby, M.W.; Bond, M.; et al. A self-management support program for older Australians with multiple chronic conditions: a randomised controlled trial. *Med J Aust* **2018,** *208*, 69-74. doi: 10.5694/mja17.00127.
25. Salisbury, C.; Man, M.S.; Bower, P.; Guthrie, B.; Chaplin, K.; Gaunt, D.M.; et al. Management of multimorbidity using a patient-centred care model: a pragmatic cluster-randomised trial of the 3D approach. *Lancet* **2018,** *392*, 41-50. doi: 10.1016/S0140-6736(18)31308-4.
26. Thorn, J.; Man, M.S.; Chaplin, K.; Bower, P.; Brookes, S.; Gaunt, D.; et al. Cost-effectiveness of a patient-centred approach to managing multimorbidity in primary care: a pragmatic cluster randomised controlled trial. *BMJ Open* **2020** *10*, e030110. doi: 10.1136/bmjopen-2019-030110.
27. Schuttner, L.; Wong, E.S.; Rosland, A.M.; Nelson, K.; Reddy, A. Association of the Patient-Centered Medical Home Implementation with Chronic Disease Quality in Patients with Multimorbidity. *J Gen Intern Med* **2020,** *35*, 2932-8. doi: 10.1007/s11606-020-06076-7.
28. Schuttner, L.; Reddy, A.; Rosland, A.M.; Nelson, K.; Wong, E.S. Association of the Implementation of the Patient-Centered Medical Home with Quality of Life in Patients with Multimorbidity. *J Gen Intern Med* **2020,** *35*, 119-25. doi: 10.1007/s11606-019-05429-1.

29. Shah, V.; Stokes, J.; Sutton, M. Effects of non-medical health coaching on multimorbid patients in primary care: a difference-in-differences analysis. *BMC Health Serv Res* **2019,** *19*, 593. doi: 10.1186/s12913-019-4367-8.
30. Sommers, L.S.; Marton, K.I.; Barbaccia, J.C.; Randolph, J. Physician, nurse, and social worker collaboration in primary care for chronically ill seniors. *Arch Intern Med* **2000,** *160*, 1825-33. doi: 10.1001/archinte.160.12.1825.
31. Swietek, K.E.; Domino, M.E.; Beadles, C.; Ellis, A.R.; Farley, J.F.; Grove, L.R.; et al. Do Medical Homes Improve Quality of Care for Persons with Multiple Chronic Conditions? *Health Serv Res,* **2018,** *53*, 4667-81. doi: 10.1111/1475-6773.13024.
32. Miranda, R.N.; Bhuiya, A.R.; Thraya, Z.; Hancock-Howard, R.; Chan, B.C.; Steele Gray, C.; et al. An Electronic Patient-Reported Outcomes Tool for Older Adults With Complex Chronic Conditions: Cost-Utility Analysis. *JMIR Aging* **2022,** *5*, e35075. doi: 10.2196/35075.
33. Stewart, M.; Fortin, M.; Brown, J.B.; Ryan, B.L.; Pariser, P.; Charles, J.; et al. Patient-centred innovation for multimorbidity care: a mixed-methods, randomised trial and qualitative study of the patients' experience. *Br J Gen Pract* **2021,** *71*, e320-e30. doi: 10.3399/bjgp21X714293.
34. Tinetti, M.; Naik, A.; Dindo, L.; Costello, D.; Esterson, J.; Geda, M.; et al. Association of Patient Priorities-Aligned Decision-Making With Patient Outcomes and Ambulatory Health Care Burden Among Older Adults With Multiple Chronic Conditions A Nonrandomized Clinical Trial. *JAMA Intern Med* **2019,** *179*, 1688-97. doi: 10.1001/jamainternmed.2019.4235.
35. Vasan, A.; Morgan, J.W.; Mitra, N.; Xu, C.; Long, J.A.; Asch, D.A.; et al. Effects of a standardized community health worker intervention on hospitalization among disadvantaged patients with multiple chronic conditions: a pooled analysis of three clinical trials. *Health Serv Res* **2020,** *55*, 894-901. doi: 10.1111/1475-6773.13321.
36. Vera, M.; Perez-Pedrogo, C.; Huertas, S.E.; Reyes-Rabanillo, M.L.; Juarbe, D.; Huertas, A.; et al. Collaborative care for depressed patients with chronic medical conditions: a randomized trial in Puerto Rico. *Psychiatr Serv* **2010,** *61*,144-50. doi: 10.1176/ps.2010.61.2.144.
37. Wakefield, B.J.; Holman, J.E.; Ray, A.; Scherubel, M.; Adams, M.R.; Hillis, S.L.; et al. Effectiveness of home telehealth in comorbid diabetes and hypertension: a randomized, controlled trial. *Telemed J E Health* **2011,** *17*, 254-61. doi: 10.1089/tmj.2010.0176.
38. Yamane, S.S.; De Gagne, J.C.; Riggs, A.; Kimberly, G.D.; Holye, M. Assessment of a patient-centered initiative to improve hypertension management for adults with comorbid type 2 diabetes at a free clinic in the rural south. *Nurs Forum* **2020,** *55*, 348-55. doi: 10.1111/nuf.12434.
39. Zamorano, P.; Muñoz, P.; Espinoza, M.; Tellez, A.; Varela, T.; Suarez, F.; et al. Impact of a high-risk multimorbidity integrated care implemented at the public health system in Chile. *PLoS One* **2022,** *17*, e0261953. doi: 10.1371/journal.pone.0261953.
