# Supplementary material for: Patient-Centred Care for Multimorbid Patients: A Scoping Review
Source: J Clin Med. 2026 May 14;15(10):3774. doi: 10.3390/jcm15103774 (PMC13207952; doi:10.3390/jcm15103774)
Supplement: Supplementary file 1 [file jcm-15-03774-s001.zip › Table_S5.pdf]

Table S5: Overview of the study characteristics.

Authors	Title	Year	Place of origin	Program	Study design	Definition multimorbidity	Setting	Age	Number of patients	Service delivery														Workforce
										Patient level								Professional level			Organizational level			
										Care plan	Goal setting	Holistic approach	Patient education	Patient participation	Self-Management support	Informal caregiver involvement	Pro-active care	Training health care providers	Patient-centered communication	Patient-centered climate	Telemedicine & Decision support	Care coordinator	Multidisciplinary team	Team members
Abadi	Achieving Whole Health: A Preliminary Study of TCMLH, a Group-Based Program Promoting Self-Care and Empowerment Among Veterans	2022	USA	Taking Charge of My Life and Health (TCMLH)	Intervention-only design	≥ 3 chronic conditions	4 Veteran Affair primary care clinics	28-80	77	x	x	x	x	x	x		x	x	x	x				TF
Ansari	Activating primary care COPD patients with multi-morbidity through tailored self-management support	2020	Australia	Activating Primary Care COPD Patients with Multimorbidity (APCOM) study	Pretest-posttest design	COPD and ≥1 co-morbidity	12 Primary care practices	40-84	50	x	x		x		x		x	x	x	x				N
Berntsen	Person-centred, integrated and pro-active care for multi-morbid elderly with advanced care needs: a propensity score-matched controlled trial	2019	Norway	Patient-Centred Team (PACT)	Prospective propensity score (PS) matched controlled trial	≥2 chronic conditions	Primary ar	>60	1218	x	x			x		x	x		x	x		x	x	NCC, PT, OT, PH, PH, MS, MD
Blom	Effectiveness and cost-effectiveness of a proactive, goal-oriented, integrated care model in general practice for older people. A cluster randomised controlled trial: Integrated Systematic Care for older People--the ISCOPE study	2016	The Netherlands	Integrated Systematic Care for older People (ISCOPE)	RCT	Problems on ≥3 health domains	59 Primary care practices	≥75	288	x	x	x		x		x	x	x					x	GP, N

Boult	Early Effects of "Guided Care" on the Quality of Health Care for Multimorbid Older Persons: A Cluster-Randomized Controlled Trial	2008	USA	Guided Care (GC)	RCT	Patients must be in the upper quartile of risk for using health services heavily during the coming year, according to their scores on the hierarchical condition category (HCC) predictive model.	8 Primary c	≥65	904	x	x		x	x	x	x	x	x	x	x	**RT**	x	x	**GP, NCC**
Boyd	Boyd CM, Reider L, Frey K, Scharfstein D, Leff B, Wollf J, et al. The effects of guided care on the perceived quality of health care for multi-morbid older persons: 18-month outcomes from a cluster-randomized controlled trial. *J Gen Intern Med.* 2010;**25**(3):235–242. doi: 10.1007/s11606-009-1192-5.	2009	USA	Guided Care (GC)	RCT	Patients must be in the upper quartile of risk for using health services heavily during the coming year, according to their scores on the HCC predictive model.	8 Primary c	≥65	904	x	x		x	x	x	x	x	x	x	x	**RT**	x	x	**GP, NCC**
Wolff	Effects of guided care on family caregivers	2010	USA	Guided Care (GC)	RCT	Patients must be in the upper quartile of risk for using health services heavily during the coming year, according to their scores on the HCC predictive model.	8 Primary c	≥65	904	x	x		x	x	x	x	x	x	x	x	**RT**	x	x	**GP, NCC**
Camacho	Long-term cost-effectiveness of collaborative care (vs usual care) for people with depression and comorbid diabetes or cardiovascular disease: a Markov model informed by the COINCIDE randomised controlled trial	2016	United Kingdom	COINCIDE	RCT	CHD and/or DMT1 or T2, with persistent depressive symptoms	36 Primary care practices	≥18	387	x	x	x	x	x	x		x	x	x	x	**RT**	x	x	**N, PWP, CC**
Coventry	Integrated primary care for patients with mental and physical multimorbidity: cluster randomised controlled trial of collaborative care for patients with depression comorbid with diabetes or cardiovascular disease	2015	United Kingdom	COINCIDE	RCT	CHD and/or DMT1 or T2, with persistent depressive symptoms	36 Primary care practices	≥18	387	x	x	x	x	x	x		x	x	x	x	**RT**	x	x	**N, PWP, CC**

del Cura-González	How to Improve Healthcare for Patients with Multimorbidity and Polypharmacy in Primary Care: A Pragmatic Cluster-Randomized Clinical Trial of the MULTIPAP Intervention	2022	Spain	MULTIPAP	RCT	≥ 3 chronic conditions and polypharmacy, defined as ≥5 drugs prescribed over the 3 months before inclusion	38 Primary care practices	65–74	593	**x**	**x**	**x**		**x**				**x**	**x**	**x**			**x**	**GP, N**
Contant	A multidisciplinary self-management intervention among patients with multimorbidity and the impact of socioeconomic factors on results	2019	Canada	PR1MaC Trial	RCT	≥3 chronic conditions	8 Primary care practices	18-75	281	**x**	**x**		**x**	**x**	**x**		**x**	**x**	**x**	**x**			**x**	**N, PT, D, T**
Fisher	Self-management program versus usual care for communitydwelling older adults with multimorbidity: A pragmatic randomized controlled trial in Ontario, Canada	2020	Canada		RCT	≥3 chronic conditions	2 Community Care Access Centers (CCAC)	≥65	59	**x**	**x**	**x**		**x**	**x**	**x**	**x**	**x**	**x**	**x**	**A&F**	**x**	**x**	**CC, N, PT, OT, HC**
Fortin	Scaling Up Patient-Centered Interdisciplinary Care for Multimorbidity: a Pragmatic Mixed-Methods Randomized Controlled Trial	2021	Canada	IMPACT plus	RCT	≥3 chronic conditions	7 Primary	18–80	284	**x**	**x**			**x**	**x**		**x**	**x**	**x**	**x**			**x**	**GP, N, D, K**
Fortin	One year follow-up and exploratory analysis of a patient-centered interdisciplinary care intervention for multimorbidity	2021	Canada	IMPACT plus	Pretest-posttest study	≥3 chronic conditions	7 Primary	18–80	284	**x**	**x**			**x**	**x**		**x**	**x**	**x**	**x**			**x**	**GP, N, D, K**
Ryan	Effect of a multimorbidity intervention on health care utilization and costs in Ontario: randomized controlled trial and propensity-matched analyses	2023	Canada	IMPACT plus	RCT	≥3 chronic conditions	9 Primary care practices	18–80	156	**x**	**x**			**x**	**x**		**x**	**x**	**x**	**x**			**x**	**GP, N, D, K**

Hochhalte	Making the Most of Your Healthcare intervention for older adults with multiple chronic illnesses	2010	USA	Making the Most of Your Healthcare intervention	RCT	≥2 of 7 qualifying chronic diseases	Primary care clinic and patients' homes	≥65	79					x							**RT**			**HC**
John	Cohort Profile: Effectiveness of a 12-Month Patient-Centred Medical Home Model Versus Standard Care for Chronic Disease Management among Primary Care Patients in Sydney, Australia	2020	Australia	WellNet	Controlled cohort study	1-3 chronic disease(s) and a risk factor or ≥4 chronic diseases	6 Primary care practices	≥40	8339	x	x	x	x	x	x		x		x	x	**RT, PP, A**	x	x	**GP, CC**
John	Outcomes of a 12-month patient-centred medical home model in improving patient activation and self-management behaviours among primary care patients presenting with chronic diseases in Sydney, Australia: a before-and-after study	2020	Australia	WellNet	Pretest-posttest study	1-3 chronic disease(s) and a risk factor or ≥4 chronic diseases	6 Primary care practices	≥40	636	x	x	x	x	x	x		x		x	x	**RT, PP, A**	x	x	**GP, CC**
Katon	Collaborative care for patients with depression and chronic illnesses	2010	USA		RCT	Depression and poorly controlled DM, CHD, or both	14 Primary	Mean age: 57.4±10.5 (Intervent ion group) - 56.3±12.1 (Control group)	214	x	x	x	x	x	x		x	x	x	x	**RT**		x	**GP, P, PWP**
Khunti	Promoting physical activity with self-management support for those with multimorbidity: a randomised controlled trial	2021	United Kingdom	The Movement through Active Personalised engagement (MAP) programme	RCT	≥2 chronic conditions	9 Primary care practices	40-85	353				x		x	x	x							**TF**
Markle-Reid	Community program improves quality of life and self-management in older adults with diabetes mellitus and comorbidity.	2018	Canada		RCT	DMT2 and ≥2 comorbidities	Diabetes education centers (DECs)	≥65	159	x		x		x	x	x	x	x	x	x		x	x	**N, D, CC, TF**

Morgan	The TrueBlue model of collaborative care using practice nurses as case managers for depression alongside diabetes or heart disease: a randomised trial	2012	Australia	TrueBlue	RCT	Depression and DMT2, CHD or both	11 Primary care practices	≥18	400	**x**	**x**	**x**		**x**	**x**		**x**	**x**	**x**	**x**		**x**	**x**	**GP, NCC**
Naik	Effect of Telephone-Delivered Collaborative Goal Setting and Behavioral Activation vs Enhanced Usual Care for Depression Among Adults With Uncontrolled Diabetes	2019	USA	Healthy Outcomes Through Patient Empowerment (HOPE)	RCT	Uncontrolled DM and depression	The Michael E. DeBakey VA Medical Center (MEDVAMC) and 6 affiliated community-based outpatient clinics	Mean age: 61.9 (±8.3)	225	**x**	**x**	**x**	**x**	**x**	**x**		**x**	**x**		**x**	**RT**			**HC**
Reed	A self-management support program for older Australians with multiple chronic conditions: a randomised controlled trial	2018	Australia	Chronic disease self management support (CDSMS) program	RCT	≥2 chronic conditions	5 Primary care practices	≥60	231	**x**	**x**	**x**		**x**	**x**		**x**			**x**	**RT**			**N/PWP**
Salisbury	Management of multimorbidity using a patient-centred care model: a pragmatic cluster-randomised trial of the 3D approach	2018	United Kingdom	The 3D approach	RCT	≥3 chronic conditions	National Health Service GP surgeries	≥18	1546	**x**	**x**	**x**		**x**			**x**	**x**	**x**	**x**	**CT**		**x**	**N, MD, PH**
Thorn	Cost-effectiveness of a patient-centred approach to managing multimorbidity in primary care: a pragmatic cluster randomised controlled trial	2020	United Kingdom	3D Intervention	RCT	≥3 chronic conditions	3 Primary care practices	≥18	1546	**x**	**x**	**x**		**x**			**x**	**x**	**x**	**x**	**CT**		**x**	**N, MD, PH**
Schuttner	Association of the Implementation of the Patient-Centered Medical Home with Quality of Life in Patients with Multimorbidity	2019	USA	Patient Aligned Care Team (PACT) initiative	Cohort study	≥2 chronic conditions	944 Veterans Health Administration clinics	≥18	22095			**x**		**x**	**x**		**x**	**x**	**x**	**x**	**MW, PP**	**x**	**x**	**GP, N, NCC, MS, PH**

Schuttner	Association of the Patient-Centered Medical Home Implementation with Chronic Disease Quality in Patients with Multimorbidity	2020	USA	the Patient-Aligned Care Team (PACT) initiative -> PACT	Cohort study	≥3 chronic diseases in > 3 body systems	917 Veterans Health Administration clinics	Veterans	318.764			**x**		**x**	**x**		**x**	**x**	**x**	**x**	**MW, PP**	**x**	**x**	**GP, N, NCC, MS, PH**
Shah	Effects of non-medical health coaching on multimorbid patients in primary care: a difference-in-differences analysis	2019	United Kingdom	"Enhanced Primary Care" model	A quasi-experimental design, a difference-in-differences analysis	≥2 chronic conditions	17 Primary care practices	≥18	3.5 million			**x**	**x**	**x**	**x**			**x**	**x**	**x**	**RT**			**HC**
Sommers	Physician, nurse, and social worker collaboration in primary care for chronically ill seniors	2000	USA	Senior Care Connections (SCC)	Controlled cohort study	≥2 chronic conditions	18 Primary care practices	≥65	543	**x**	**x**		**x**	**x**	**x**	**x**	**x**	**x**		**x**	**RT**		**x**	**GP, N, SW**
Swietek	Do Medical Homes Improve Quality of Care for Persons with Multiple Chronic Conditions?	2018	USA	Community Care of North Carolina (CCNC)	non-RCT	At least two of eight target conditions	1600 Prima	18-64	131.036			**x**	**x**	**x**			**x**					**x**		**CC**
Miranda	An Electronic Patient-Reported Outcomes Tool for Older Adults With Complex Chronic Conditions: Cost-Utility Analysis	2022	Canada	ePRO tool	RCT	≥2 chronic conditions	6 Primary c	≥60	45	**x**	**x**			**x**	**x**	**x**	**x**				**PP**			**-**
Stewart	Patient-centred innovation for multimorbidity care: a mixed-methods, randomised trial and qualitative study of the patients' experience	2021	Canada	the Telemedicine IMPACT Plus intervention	RCT	≥3 chronic conditions	9 Primary care practices	18-80	163	**x**	**x**			**x**			**x**				**RV**	**x**	**x**	**GP, MD, P, N, SW, PT, OT, PH, D, CC**
Tinetti	Association of Patient Priorities-Aligned Decision-Making With Patient Outcomes and Ambulatory Health Care Burden Among Older Adults With Multiple Chronic Conditions: A Nonrandomized Clinical Trial	2019	USA	/	non-RCT	≥3 chronic conditions	1 Primary care practice	≥65	366		**x**			**x**				**x**	**x**	**x**	**RT**	**x**	**x**	**GP, N, CC**

Vasan	Effects of a standardized community health worker intervention on hospitalization among disadvantaged patients with multiple chronic conditions: a pooled analysis of three clinical trials	2020	USA	IMPaCT	RCT	≥2 chronic conditions	Primary ar	18-64	446 (Trial 1) - 302 (Trial 2) - 592 (Trial 3)	**x**	**x**	**x**		**x**		**x**	**x**	**x**	**x**	**x**		**x**		**CC**
Vera	Collaborative care for depressed patients with chronic medical conditions: a randomized trial in Puerto Rico	2010	Puerto Rico	/	RCT	major depression and chronic general medical conditions	14 Primary care practices	≥18	179				**x**	**x**				**x**			**RT**	**x**	**x**	**MD, PWP, CC**
Wakefield	Effectiveness of home telehealth in comorbid diabetes and hypertension: a randomized, controlled trial	2011	USA	/	RCT	DM and hypertension	Iowa City VA Medical Center (ICVAMC)	Veterans	304				**x**	**x**	**x**		**x**				**TD**	**x**		**NCC**
Yamane	Assessment of a patient-centered initiative to improve hypertension management for adults with comorbid type 2 diabetes at a free clinic in the rural south	2020	USA	/	Pretest-posttest study	DMT2 and hypertension	A free primary care clinic	≥18	24	**x**			**x**	**x**	**x**		**x**	**x**	**x**	**x**	**DA**			**N**
Zamorano	Impact of a high-risk multimorbidity integrated care implemented at the public health system in Chile	2022	Chile	Multimorbidity patient-centered care model (MPCM)	Cohort study	≥2 chronic conditions	14 Primary health care centers	>15	3.933	**x**	**x**	**x**	**x**	**x**	**x**		**x**	**x**	**x**	**x**	**RT**	**x**	**x**	**GP, NCC, N, M, D, PWP**

R = remote communication: telephone (RT) or video (RV). A&F = Audit & Feedback system. A = an application which enables sharing of a range of health resources including video series, links to credible websites, apps, and tools tailored to patients' information needs. CT = an interactive computerized template enabled sharing of data between the clinicians. PP = internet-based patient portal, which allows patients to manage prescriptions and view test results and appointments. MW = a secure messaging website, which allows patients to send electronic messages to their team. TD = a home telehealth device, which allows patients to enter measurements. DA = decision aid. MD = medical doctor. S = specialist. GP = general practitioner. N = nurse. NCC = nurse care coordinator. CC = care coordinator. PT = physiotherapist. OT = occupational therapist.

HC = health coach. TF = (non-medical) trained facilitator. PH = pharmacist. P = psychiatrist. MS = medical secretary. PWP = Psychological Well Being Practitioner or psychologist. D = dietitian. T = tobaccoologist. K = kinesiologists. SW = social worker. M = midwives.
